# Associations between smoking and caffeine consumption in two European cohorts

**DOI:** 10.1111/add.13298

**Published:** 2016-03-27

**Authors:** Jorien L. Treur, Amy E. Taylor, Jennifer J. Ware, George McMahon, Jouke‐Jan Hottenga, Bart M. L. Baselmans, Gonneke Willemsen, Dorret I. Boomsma, Marcus R. Munafò, Jacqueline M. Vink

**Affiliations:** ^1^ Department of Biological PsychologyVU University AmsterdamAmsterdamthe Netherlands; ^2^ EMGO+ Institute for Health and Care ResearchVU University Medical CenterAmsterdamthe Netherlands; ^3^ UK Centre for Tobacco and Alcohol Studies, School of Experimental PsychologyUniversity of BristolBristolUK; ^4^ MRC Integrative Epidemiology Unit at the University of BristolBristolUK; ^5^ School of Social and Community MedicineUniversity of BristolBristolUK; ^6^ Neuroscience Campus AmsterdamVU University Medical CenterAmsterdamthe Netherlands

**Keywords:** ALSPAC, caffeine, coffee, NTR, smoking

## Abstract

**Aims:**

To estimate associations between smoking initiation, smoking persistence and smoking heaviness and caffeine consumption in two population‐based samples from the Netherlands and the United Kingdom.

**Design:**

Observational study employing data on self‐reported smoking behaviour and caffeine consumption.

**Setting:**

Adults from the general population in the Netherlands and the United Kingdom.

**Participants:**

Participants from the Netherlands Twin Register [NTR: *n* = 21 939, mean age 40.8, standard deviation (SD) = 16.9, 62.6% female] and the Avon Longitudinal Study of Parents and Children (ALSPAC: *n* = 9086, mean age 33.2, SD = 4.7, 100% female).

**Measurements:**

Smoking initiation (ever versus never smoking), smoking persistence (current versus former smoking), smoking heaviness (number of cigarettes smoked) and caffeine consumption in mg per day through coffee, tea, cola and energy drinks.

**Findings:**

After correction for age, gender (NTR), education and social class (ALSPAC), smoking initiation was associated with consuming on average 52.8 [95% confidence interval (CI) = 45.6–60.0; NTR] and 59.5 (95% CI = 51.8–67.2; ALSPAC) mg more caffeine per day. Smoking persistence was also associated with consuming more caffeine [+57.9 (95% CI = 45.2–70.5) and +83.2 (95% CI = 70.2–96.3) mg, respectively]. Each additional cigarette smoked per day was associated with 3.7 (95% CI = 1.9–5.5; NTR) and 8.4 (95% CI = 6.9–10.0; ALSPAC) mg higher daily caffeine consumption in current smokers. Smoking was associated positively with coffee consumption and less strongly with cola and energy drinks. For tea, associations were positive in ALSPAC and negative in NTR.

**Conclusions:**

There appears to be a positive association between smoking and caffeine consumption in the Netherlands and the United Kingdom.

## Introduction

Cigarette smoking is associated with higher consumption of coffee 1. In a sample of individuals from the United States who never drank coffee, 4.8% of males and 8.1% of females were smokers, compared with 34.7% and 48.1%, respectively, in those who drank six or more cups of coffee per day 2. A positive correlation (*r* = 0.13) was also reported between number of cigarettes smoked and level of total caffeine consumption among British smokers 3. Less is known about the association between smoking and the consumption of caffeinated drinks other than coffee. Klesges *et al.* 4 found no differences in smoking behaviour between (caffeinated) tea drinkers and non‐drinkers in a US‐based sample. In two studies linking adolescent consumption of energy drinks to health behaviours, regular consumption of energy drinks (≥ 1 week) was associated with a higher odds of smoking initiation 5 and frequency of energy drink use was correlated positively (*r* ~ 0.2) with past 30‐day frequency of cigarette use 6. Terry‐McElrath *et al*. 6 also found positive correlations of 0.12–0.23 between soft drink consumption and cigarette use, but no distinction was made between cola (which contains caffeine) and other soft drinks (which generally do not contain caffeine).

Preferences for type of caffeinated drink, in particular coffee versus tea, vary between countries. Most of the available literature on the association between smoking and caffeine use is based on populations from the United States. The current study includes two European cohorts, one of which is Dutch and one is British. While tea is the dominant drink in the United Kingdom 7, the Dutch are reportedly among the world's heaviest coffee drinkers 8. Such cultural differences may have an influence on the association between smoking behaviour and caffeine consumption, but so far this has not been investigated. Smoking prevalence does not differ greatly between the two countries with 26% of Dutch men and 20% of Dutch women being smokers in 2013 9, compared with 22% and 17% of British men and women, respectively, in that same year 10.

The popularity of caffeine as a psychoactive substance, and the high burden of morbidity and mortality due to smoking, make it important to understand their relationship more clearly. In order to achieve this aim, it is necessary to first explore in depth the associations among different aspects of smoking behaviour and the use of different types of caffeinated drinks. This will help to determine the importance of the relationship between smoking and caffeine and guide the development of future intervention or preventive studies. In this study, associations between self‐reported smoking behaviour and caffeine consumption are investigated in the Netherlands Twin Register (NTR) (*n* = 21 939, the Netherlands) and the Avon Longitudinal Study of Parents and Children (ALSPAC) (*n* = 9086, United Kingdom). Data from these two samples are analysed separately, so that any cultural differences in the association between smoking and caffeine can be distinguished. There were two main research questions: (1) is the association between smoking and caffeine consumption consistent across different types of caffeinated drinks or is it specific to coffee; and (2) is the association between smoking and caffeine consumption consistent across two European countries with different patterns of caffeine consumption?

## Methods

### Study sample

Participants of two large population‐based studies were included: the NTR and the ALSPAC.

The NTR is an ongoing longitudinal study of Dutch twins and their family members, which was established in 1987. Adolescent and young adult twins were recruited initially through city council offices across the Netherlands. Over time, recruitment of additional twins and family members of twins (among which parents, siblings and spouses) has continued through several approaches. A fuller description of this population‐based study and its design can be found elsewhere 11. A total of 17 998 adult participants who completed the 10th survey of the NTR in 2013–14 were included in the present investigation. This survey contained, among others, questions on smoking and the consumption of an extensive list of caffeinated and decaffeinated drinks. An additional 2896 individuals were selected who did not participate in the 10th survey but completed the 5th NTR survey in 2000, which included questions on coffee consumption. Data from 60 additional individuals were added because they participated in a brief online survey focused specifically on coffee consumption, sent out in 2012. Adding these three samples resulted in a total group of 21 939 individuals [mean age 40.8, standard deviation (SD) = 16.9, 62.6% female].

ALSPAC is a prospective cohort study into which 14 541 pregnant women residing in the county of Avon in the United Kingdom with expected delivery dates ranging between 31 December 1992 and 1 April 1999 were recruited. Ethics approval for the study was obtained from the ALSPAC Ethics and Law Committee and the Local Research Ethics Committees. Since recruitment, regular follow‐ups have been conducted consisting of self‐report surveys and clinic visits of the mothers, their children and their partners. An extensive description of this study and its methods is available elsewhere 12, 13. Surveys containing questions on smoking and caffeinated and decaffeinated coffee, tea and cola consumption were sent to the mothers during pregnancy at 18 and 32 weeks gestation, and after delivery when the child was aged 2, 47, 85, 97 and 145 months. Data at all time‐points were analysed, but only the results from the time that the child was 47 months are presented (*n* = 9086, mean age mothers 33.2, SD = 4.7, 100% female). This specific time‐point was selected because of its large sample size and because smoking behaviour and caffeine use may be different during and immediately after pregnancy. To check if temporary, pregnancy‐related changes in smoking and caffeine use affected their association, data from all time‐points were analysed (see online Supporting information). The study website contains details of all the data that are available through a fully searchable data dictionary (www.bris.ac.uk/alspac/researchers/data‐access/data‐dictionary).

### Smoking behaviour

Participants were classified as current smokers, former smokers or never smokers. In the NTR sample, smoking status was based on two questions: ‘Have you ever smoked?’, with answer categories ‘no’, ‘a few times just to try’ and ‘yes’, and ‘How often do you smoke now?’, with answer categories ‘I don't smoke regularly’, ‘I've quit smoking’, ‘once a week or less’, ‘a few times a week’ and ‘once a day or more’. Those who said ‘yes’ when asked the first question and stated they currently smoked once a week or more were classified as current smokers, while those who said ‘I've quit smoking’ to the second question were classified as former smokers. When answers to these two questions were contradictory or missing, additional questions such as ‘How many years have you smoked?’ or ‘At what age did you quit smoking?’ determined classification. Current smokers were asked how many cigarettes they smoke on average per day (for daily users) or per week (for weekly users). In the ALSPAC sample, smoking status was determined by the open‐ended question: ‘About how many cigarettes do you smoke each day?’. Participants were classified as smokers when they reported smoking one cigarette per day or more (there was no question on weekly use). An individual was identified as a former smoker when they had reported smoking at one of the previous time‐points, but reported not smoking in the current survey. Participants who said they smoked zero cigarettes per day and had not reported previously that they were smoking were classified as never smokers.

### Caffeine consumption

In both samples, questions were asked about caffeinated coffee, tea (including green tea in NTR) and cola, while in the NTR an additional question on energy drinks was included. NTR participants were asked whether they drank each of these drinks daily, weekly or (almost) never. The average number of drinks per day (for daily use) or per week (for weekly use) was also obtained. In the case of weekly use, the number of drinks was divided by seven to obtain an estimate of average daily use. ALSPAC participants were asked how many cups of coffee and tea they currently drank (open format) separately for weekdays and weekends. The replies to these questions were recoded into one measure of daily use. For cola, there was a closed format question on number of drinks per week (‘never or rarely’, ‘once every 2 weeks’, ‘1–3 times a week’, ‘4–7 times a week’, ‘once a day or more’), which was recoded to, respectively, 0, 0.5, 2, 5.5 and 7 per week. For all questions it was made clear that participants should report on caffeinated drinks only. Caffeine use was computed by weighting the drinks by their caffeine content. Caffeine content (in mg per serving) was set at 75 mg per cup of regular coffee, 65 mg per cup of espresso (only in NTR), 40 mg per cup of regular tea, 20 mg per cup of green tea (only in NTR), 10 mg per 100 ml of cola (a serving was specified as one can of 330 ml in ALSPAC and as one glass of 180 ml in NTR) and 80 mg per can of energy drink (only in NTR) 14. For an estimate of total daily caffeine use, the daily caffeine intake of all drinks was summed.

### Statistical analyses

All regression analyses were performed in Stata version 9.0 (StataCorp LP, College Station, TX, USA) and corrected for family clustering by utilizing the robust cluster option in NTR data. This function takes information on family relatedness and uses it to correct for the correlation within families (i.e. clusters). Linear regression analyses were performed with daily caffeine consumption (mg per day) as the dependent variable. The independent variable was either smoking initiation (0 = never smoking, 1 = ever smoking) or smoking persistence (0 = former smoking, 1 = current smoking). Associations between smoking behaviour and the consumption of decaffeinated coffee (dichotomized into 0 = non‐users, 1 = users) were also tested.

The association between cigarettes smoked per day (independent variable) and daily caffeine consumption (dependent variable) was investigated using linear regression analyses. For these analyses, non‐smokers and non‐caffeine users (or non‐users of a specific drink when analysed individually) were excluded. This was to test whether an increase in cigarettes smoked per day is associated with an increase in caffeine consumption in those who consumed at least some caffeine to start with.

All analyses described here were performed for total caffeine use, individual caffeinated drinks and decaffeinated coffee, both unadjusted and adjusted for age, educational attainment [ALSPAC in five categories: secondary education (CSE), vocational, O level, A level and Degree; NTR in seven categories: primary school only, lower vocational schooling, lower secondary schooling, intermediate vocational schooling, intermediate/higher secondary schooling, higher vocational schooling and university], social class [only in ALSPAC in six categories: class I, class II, class II (non‐manual), class III (manual), class IV and class V] and gender (only in NTR).

## Results

### Descriptive statistics

Table 1 depicts smoking status and daily caffeine use for 21 939 male and female NTR participants and 9086 female ALSPAC participants. There were more current smokers in the British sample (22.9%) compared with the Dutch sample (14.9% for women and 17.6% for men), and the number of cigarettes smoked per day was higher in the British women (mean 12.6) compared with the Dutch smokers (10.6 in women and 11.4 in men). Men were more likely to be coffee drinkers (81.9%) than women (67.0% in Dutch women and 59.0% in British women), while for tea the opposite was true (60.9% in Dutch men compared to 75.5% in Dutch women and 78.4% in British women). On average, the Dutch consumed more coffee per day (in the total group, 2.2 cups in women and 3.8 cups in men) than the British women (1.8 cups). For tea, a higher consumption was found in the British (mean of 3.0 cups) compared with the Dutch participants (mean of 2.1 cups for women and 1.3 for men). Daily or weekly cola use was more common in the British (51.4%) compared with the Dutch sample (26.1% for women and 36.7% for men), while energy drinks were rarely consumed on a daily or weekly basis (4.4% in Dutch women and 5.9% in Dutch men). Any consumption of decaffeinated coffee ranged from 8.9 to 18.8%.

**Table 1:** Descriptive statistics on smoking behaviour and caffeine use in the Netherlands Twin Register (NTR) and the Avon Longitudinal Study of Parents and Children (ALSPAC).

			*NTR*		*ALSPAC*
			*Men*	*Women*	*Women*
Age (years)		Mean years (SD)	42.3 (17.5)	39.9 (16.5)	33.2 (4.7)
Smoking status		Never smoker (*n*, %)	4463 (54.5%)	8579 (62.4%)	4966 (54.7%)
		Former smoker (*n*, %)	2289 (27.9%)	3120 (22.7%)	2035 (22.4%)
		Current smoker (*n*, %)	1440 (17.6%)	2048 (14.9%)	2085 (22.9%)
Number of cigarettes per day in smokers		Mean (SD)	11.4 (7.6)	10.6 (7.2)	12.6 (6.9)
Any caffeine use		(*n*, %)	7541 (97.2%)	12 189 (95.9%)	8339 (95.0%)
Daily caffeine use total	in total group	Mean mg (SD)	334.4 (216.9)	240.0 (179.7)	260.9 (170.9)
Daily caffeine use total	in users	Mean mg (SD)	346.1 (211.3)	251.7 (175.9)	274.6 (164.2)
Any coffee use		(*n*, %)	6622 (81.9%)	9106 (67.0%)	5318 (59.0%)
Daily coffee use	in total group	Mean mg (SD)	280.2 (222.7)	166.3 (174.2)	135.3 (164.9)
		Mean cups (SD)	3.8 (3.0)	2.2 (2.3)	1.8 (2.2)
	in users	Mean mg (SD)	342.0 (198.5)	248.3 (157.9)	229.2 (156.7)
		Mean cups (SD)	4.6 (2.7)	3.3 (2.1)	3.1 (2.1)
Any tea use		(*n*, %)	3946 (60.9%)	8498 (75.5%)	7044 (78.4%)
Daily tea use	in total group	Mean mg (SD)	46.2 (68.4)	65.9 (81.3)	121.5 (106.0)
		Mean cups (SD)	1.3 (1.9)	2.1 (2.5)	3.0 (2.7)
	in users	Mean mg (SD)	75.8 (73.7)	87.3 (83.0)	155.1 (95.5)
		Mean cups (SD)	2.2 (2.0)	2.8 (2.5)	3.9 (2.4)
Any cola use		*n* (%)	2375 (36.7%)	2933 (26.1%)	4596 (51.4%)
Daily cola use	in total group	Mean mg (SD)	6.1 (14.4)	3.9 (11.8)	4.7 (7.6)
		Mean servings (SD)	0.3 (0.8)	0.2 (0.7)	0.1 (0.2)
	in users	Mean mg (SD)	16.6 (19.7)	14.9 (19.3)	9.1 (8.5)
		Mean servings (SD)	0.9 (1.1)	0.8 (1.1)	0.3 (0.3)
Any energy drink use		(*n*, %)	379 (5.8%)	500 (4.4%)	–
Daily energy drink use	in total group	Mean mg (SD)	2.1 (12.7)	1.8 (14.3)	–
		Mean servings (SD)	0.03 (0.2)	0.02 (0.2)	–
	in users	Mean mg (SD)	35.4 (39.9)	41.1 (54.4)	–
		Mean servings (SD)	0.4 (0.5)	0.5 (0.7)	–
Any decaffeinated coffee use		(*n*, %)	575 (8.9%)	1225 (10.9%)	1697 (18.8%)
Daily decaffeinated coffee use	in total group	Mean cups (SD)	0.2 (0.9)	0.2 (0.8)	0.5 (1.3)
	in users	Mean cups (SD)	2.3 (2.1)	2.0 (1.7)	2.5 (1.8)

Participants with missing gender (*n* = 7, only in NTR) were excluded from this table. Any use = weekly or daily use. SD = standard deviation.

Strong associations were found between smoking/caffeine use and educational attainment, social class and age (see Table 2). In the Dutch sample, older participants were more likely to have initiated smoking, while their odds of smoking persistence (being a current smoker rather than a former smoker) were lower. In the British sample, older age was associated with lower odds of both smoking initiation and smoking persistence. Older participants consumed more total caffeine, coffee and tea, but they consumed less cola and energy drinks. Participants with a higher educational attainment or a higher social class were less likely to have initiated smoking and less likely to still be smoking when smoking was initiated (smoking persistence). Higher educational attainment and social class were also associated with a lower consumption of all caffeinated drinks, except for tea in the Dutch sample where a higher education was associated with a higher consumption. These variables (age, gender, educational attainment and social class) were included in all analyses as covariates.

**Table 2:** Associations between covariates and smoking initiation, smoking persistence and daily caffeine consumption (in mg) in the Netherlands Twin Register (NTR) and the Avon Longitudinal Study of Parents and Children (ALSPAC).

		*Smoking initiation – OR (95% CIs)*		*Smoking persistence – OR (95% CIs)*		*Total caffeine – β (95% CIs)*		*Coffee – β (95% CIs)*		*Tea – β (95% CIs)*		*Cola – β -(95% CIs)*		*Energy drink– β (95% CIs)*
*NTR*	*n*		*n*		*n*		*n*		*n*		*n*		*n*	
Age (years)	21 843	1.04 (1.03 to 1.04)	8849	0.94 (0.94 to 0.94)	17 640	4.6 (4.4 to 4.8)	21 583	4.0 (3.9 to 4.2)	17 640	0.4 (0.4 to 0.5)	17 640	−0.1 (−0.1 to −0.1)	17 640	−0.1 (−0.1 to −0.1)
Gender	21 939	0.72 (0.68 to 0.76)	8897	1.04 (0.96 to 1.14)	17 731	−94.5 (−100.4 to −88.5)	21 677	−113.8 (−119.2 to −108.5)	17 731	19.7 (17.4 to 22.1)	17 731	−2.2 (−2.6 to −1.8)	17 731	−0.2 (−0.7 to 0.2)
Education	14 816	0.55 (0.51 to 0.59)	6752	0.77 (0.69 to 0.85)	11 851	−14.6 (−21.6 to −7.6)	14 632	−28.1 (−34.7 to −21.5)	11 851	11.0 (8.1 to 13.9)	11 851	−0.2 (−0.6 to 0.3)	11 851	−0.4 (−0.7 to −0.1)
*ALSPAC*	*n*		*n*		*n*		*n*		*n*		*n*		*n*	
Age	8960	0.96 (0.95 to 0.97)	4062	0.91 (0.90 to 0.92)	8864	1.0 (0.2 to 1.7)	9095	0.4 (−0.3 to 1.1)	9080	0.9 (0.4 to 1.3)	9026	−0.3 (−0.4 to −0.3)	*–*	–
Education	8798	0.58 (0.53 to 0.64)	3970	0.45 (0.40 to 0.52)	8721	−15.1 (−22.4 to −7.7)	8942	−7.5 (−14.5 to −0.5)	8929	−4.3 (−8.8 to 0.2)	8883	−2.0 (−2.4 to −1.7)	*–*	–
Social class	7442	0.77 (0.70 to 0.84)	3304	0.61 (0.53 to 0.71)	7371	−3.0 (−10.8 to 4.7)	7542	−1.2 (−8.7 to 6.3)	7538	−0.6 (−5.3 to 4.2)	7498	−1.5 (−1.8 to −1.1)	*–*	–

Regression analyses were performed with smoking initiation, smoking persistence, total caffeine use, coffee, tea, cola or energy drink as the dependent variable and age (continuous), gender (0 = male 1 = female), education (0 = low 1 = high) or social class (only in ALSPAC: 0 = low 1 = high) as the independent variable. For NTR low education = primary school only, lower vocational schooling, lower secondary schooling, intermediate vocational schooling or intermediate/higher secondary schooling and high education = higher vocational schooling or university; for ALSPAC low education = secondary education (CSE), vocational or O level and high education = A level or Degree. Low social class = class II (non‐manual), class III (manual), class IV or class V and high social class = class I or class II.

### Smoking initiation and caffeine use

**Figure 1:**
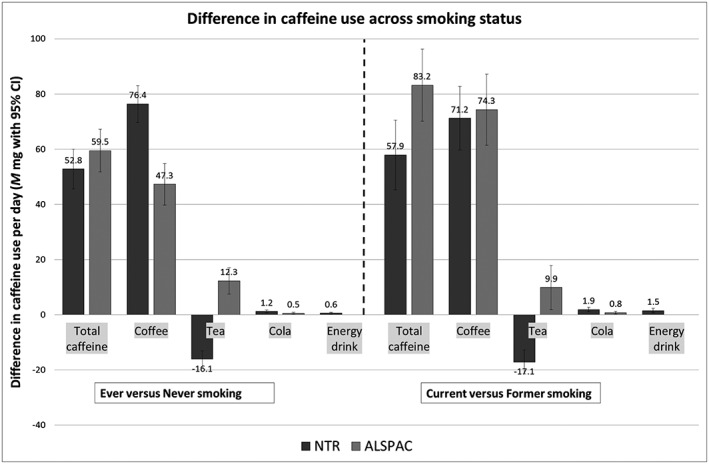
NTR = Netherlands Twin Register; ALSPAC = Avon Longitudinal Study of Parents and Children. For ever versus never smoking the number of participants for each analysis in the NTR was 11 850 for total caffeine, 14 564 for coffee, 11 805 for tea, 11 805 for cola and 11 805 for energy drinks and in ALSPAC, 7117, 7278, 7277, 7233 and 0, respectively. For current versus former smoking the number of participants in the NTR was 5400 for total caffeine, 6619 for coffee, 5400 for tea, 5400 for cola and 5400 for energy drinks and in ALSPAC 3155, 3226, 3233, 3210 and 0, respectively

In both the Dutch and the British sample, ever smokers consumed more caffeine than never smokers [respectively, +52.8 mg, 95% confidence interval (CI) = 45.6–60.0) and +59.5 mg, 95% CI = 51.8–67.2, Fig. 1, left‐hand side]. The same pattern was seen when exploring coffee use only. While Dutch ever smokers consumed less tea compared with never smokers (−16.1, 95% CI = –19.1 to −13.1), results were opposite in the British sample (+12.3, 95% CI = 7.5–17.1). Ever smokers consumed slightly more cola and energy drinks compared with never smokers (ranging from +0.5, 95% CI = 0.2–0.9 to +1.2, 95% CI = 0.8–1.7).

### Smoking persistence and caffeine use

Current smokers consumed more coffee, cola and energy drinks than former smokers, resulting in a higher overall caffeine intake (Fig. 1, right‐hand side). Again, contrasting results were found for the consumption of tea, with Dutch current smokers using less (−17.1, 95% CI = –21.5 to −12.7), while British smokers used more tea compared to former smokers (+9.9, 95% CI = 1.9–17.9).

### Smoking heaviness and caffeine use in current smokers

**Figure 2:**
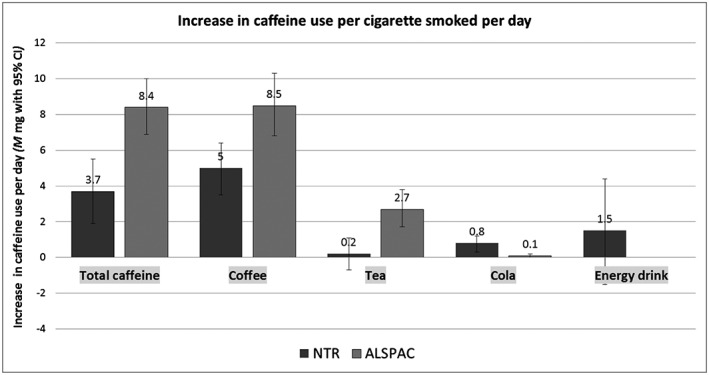
NTR = Netherlands Twin Register; ALSPAC = Avon Longitudinal Study of Parents and Children. For the NTR the number of participants for each analysis was 1282 for total caffeine, 1790 for coffee, 717 for tea, 402 for cola and 50 for energy drinks while for ALSPAC these numbers were 1408, 993, 1125, 877 and 0, respectively

Each cigarette smoked per day was associated with an increased consumption of 3.7 mg (95% CI = 1.9–5.5) caffeine in the Dutch and 8.4 (95% CI = 6.9–10.0) in the British sample (Fig. 2). Number of cigarettes per day was also associated positively with caffeine use through coffee and cola. For total caffeine and coffee, the effect was stronger in the British sample compared to the Dutch sample. While British smokers consumed more tea with every additional cigarette (+2.7 mg, 95% CI = 1.7–3.8), there was no association in Dutch smokers (+0.2 mg, 95% CI = –0.7 to 1.1). When grouping smoking heaviness into categories of < 5, 5–9, 10–14, 15–19, 20–24 and 25+ cigarettes per day, a linear association was seen with total caffeine and coffee in the NTR (Supporting information, Fig. S1) and with total caffeine, coffee and tea in ALSPAC (Supporting information, Fig. S2). Due to the weaker association between number of cigarettes per day and cola use, linearity was less distinct when grouping smoking heaviness into categories. For ALSPAC, results at time‐points other than the one described here (at 47 months after delivery) were very similar.

Smoking heaviness was not associated with the consumption of decaffeinated coffee in ALSPAC, while smoking persistence and, at some time‐points, smoking initiation was associated with lower odds of consuming decaffeinated coffee (Supporting information, Table S4‐S6). In the NTR current smokers had lower odds of decaffeinated coffee use compared with former smokers (Supporting information, Table S10‐S12).

## Discussion

Smoking behaviour was associated positively with caffeine consumption in two large population‐based samples, with evidence for cultural differences in the relationship between smoking and tea. This is the first time that the consumption of coffee, tea, cola and energy drinks and the association with smoking behaviour was investigated comparing data from two different countries: a ‘coffee drinking’ country (the Netherlands) and a ‘tea‐drinking’ country (the United Kingdom).

The British participants of ALSPAC drank more tea than the Dutch participants of the NTR. While the Dutch drank more coffee than the British, this difference was less distinct. These findings, based on self‐report, are in agreement with comparisons between the Netherlands and the United Kingdom based on historical and sales figures 7, 8. For total caffeine use, there was a strong and positive association with smoking initiation, smoking persistence and smoking heaviness. Similar associations were found when assessing coffee separately, consistent with earlier findings in populations from the United States 2, the United Kingdom 3 and the Netherlands 15.

The first research question was whether the association between smoking and caffeine consumption is consistent across different types of caffeinated drinks, or specific to coffee. Smoking initiation and smoking persistence were associated with consuming more tea in the British sample, while the opposite was true in the Dutch sample. These results were not altered substantially after excluding green tea in the Dutch sample. Associations between smoking and tea were also largely the same when including coffee as a covariate as were smoking–coffee associations when including tea as a covariate (data not shown). A possible explanation for the cultural difference for tea is that its consumption is very common in the United Kingdom, while in the Netherlands tea drinkers differ from non‐drinkers. This explanation is supported by the fact that in the Dutch sample higher educational attainment was associated with drinking more tea, while in the British sample neither education nor social class were associated with tea consumption. All analyses were corrected for these variables, but it may be that there were other, unmeasured covariates that made Dutch tea drinkers different from British tea drinkers. For instance, in a population‐based cohort of ~40 000 Dutch individuals, higher tea consumption (ranging from 0 to 1 cups per day to > 5 cups per day) was associated not only with a lower prevalence of current smoking, but also with a reduction in alcohol consumption, body mass index (BMI) and total energy intake. In contrast, higher coffee consumption was accompanied by a higher prevalence of current smoking and higher alcohol consumption, BMI and total energy intake 15. High correlations between smoking, alcohol and cannabis have been reported previously 16, 17, 18. Our findings in Dutch and British individuals are in contrast with a previous US‐based study that found no difference in smoking behaviour between tea drinkers and non‐tea‐drinkers 4. This could be due to differences in the social patterning of tea use across these countries. In the US‐based study, male sex, older age and higher educational attainment were associated with lower odds of being a (caffeinated) tea drinker. In contrast, in our analyses older age and, in the Dutch sample, higher educational attainment were associated with drinking more tea. These findings emphasize the need to study such behaviours in multiple (culturally distinct) populations. Small but consistent positive associations were also found between cola/energy drink consumption and smoking initiation, smoking persistence and (for cola only) smoking heaviness. This supports previous research linking smoking to a higher consumption of soft drinks 6 and energy drinks 5 in adolescents. We have now replicated these findings for energy drinks and cola (which is caffeinated) specifically, and have shown that it also applies to an adult population.

Overall, our findings suggest a strong association between smoking and the most commonly used caffeinated drinks. Except for tea, this association was consistent across the Netherlands and the United Kingdom answering the second research question. In the NTR the prevalence of current smoking was lower than in the general Dutch population. This (slight) bias is due probably to a relatively high proportion of highly educated participants 19. These differences were accounted for by correcting all analyses for educational attainment and in ALSPAC also for social class.

This study has some limitations that need to be considered. Most importantly, the two included samples are not entirely comparable and may not be generalizable to other populations. The Dutch sample comprises men and women from twin families, while the British sample consists of women only. By comparing less cooperative to highly cooperative families, the Dutch sample has found previously that data on health, personality and life‐style were only mildly biased by non‐response 19. In the present study we corrected for age, gender, educational attainment and social class to minimize possible bias. Regarding the ALSPAC sample, it may be that mothers of young children adjust their smoking and/or caffeine use during or after pregnancy. For this reason, and because of sample size, we analysed data from 47 months after delivery. When analysing all time‐points between 18 weeks gestation and 145 months after delivery, there were no major differences in the association between smoking and caffeine use. Also, our findings were not altered substantially when comparing the (female) ALSPAC sample to female NTR participants only, instead of including both male and female NTR participants and correcting for gender (data not shown). In line with previous studies of NTR and ALSPAC, we defined regular smoking as (minimally) weekly smoking for the NTR compared to daily smoking for ALSPAC. To check whether this discrepancy affected the comparability of our findings we re‐analysed the NTR data such that only daily smoking was identified in both samples. The results of these analyses were not substantially different (data not shown).

Different mechanisms have been suggested to explain the strong association between smoking and caffeine. From previous work we know that both smoking and caffeine use are influenced moderately to strongly by genetic factors 20, 21. It could therefore be that smoking and caffeine are associated because they are influenced by the same genes. Evidence for shared genetic and environmental factors between smoking and caffeine use was indeed found in two US‐based twin studies 22, 23, 24, 25. The subtle cultural differences found in the present study emphasize the need for bivariate twin studies in other populations. A second explanation for the association between smoking and caffeine is a causal effect from caffeine use on smoking or vice versa. Experimental work in animal and human subjects has found evidence for causal effects in both directions. Smoking may increase caffeine use causally because nicotine in inhaled cigarette smoke increases the metabolism of caffeine 26, 27, 28. In the other direction, it was found that rats consuming caffeine in their drinking water self‐administered significantly more nicotine than did controls. It was hypothesized that this was due to a pharmacokinetic interaction such that caffeine potentiates the reinforcing properties of nicotine 29, 30, 31. Causal effects need to be studied further, with one way of assessing causality being Mendelian randomization analysis (MR). This technique utilizes genetic variants associated with a certain trait as an instrument, or proxy, for that trait, thereby minimizing effects of confounding and reverse causation 32. To gain further insight into the association between smoking and caffeine, additional research utilizing the genetically informative methods described above and newly emerging methods is required. Here, we have identified an association between smoking behaviour and caffeine use. If this association is due (partly) to causal effects, there could be important implications. For instance, knowledge of factors that causally increase or decrease the odds of quitting smoking would be valuable, as many smokers who attempt to quit fail 33, 34.

## Declaration of interests

None.

## Supporting information


**Table S1** Associations between smoking initiation (ever versus never smokers) and daily caffeine consumption (in mg) in the Avon Longitudinal Study of Parents and Children (ALSPAC).
**Table S2**. Associations between smoking persistence (current versus former smokers) and daily caffeine consumption (in mg) in the Avon Longitudinal Study of Parents and Children (ALSPAC).
**Table S3**. Associations between number of cigarettes smoked per day and daily caffeine consumption (in mg) in smokers from the Avon Longitudinal Study of Parents and Children (ALSPAC).
**Table S4**. Associations between smoking initiation (ever versus never smokers) and decaffeinated coffee consumption (users versus non‐users) in the Avon Longitudinal Study of Parents and Children (ALSPAC).
**Table S5**. Associations between smoking persistence (current versus former smokers) and decaffeinated coffee consumption (users versus non‐users) in the Avon Longitudinal Study of Parents and Children (ALSPAC).
**Table S6.** Associations between number of cigarettes smoked per day and decaffeinated coffee consumption (users versus non‐users) in smokers from the Avon Longitudinal Study of Parents and Children (ALSPAC).
**Table S7**. Associations between smoking initiation (ever versus never smokers) and daily caffeine consumption (in mg) in the Netherlands Twin Register (NTR).
**Table S8.** Associations between smoking persistence (current versus former smokers) and daily caffeine consumption (in mg) in the Netherlands Twin Register (NTR).
**Table S9.** Associations between number of cigarettes smoked per day and daily caffeine consumption (in mg) in smokers from the Netherlands Twin Register (NTR).
**Table S10.** Associations between smoking initiation (ever versus never smokers) and decaffeinated coffee consumption (users versus non‐users) in the Netherlands Twin Register (NTR).
**.** Associations between smoking persistence (current versus former smokers) and decaffeinated coffee consumption (users versus non‐users) in the Netherlands Twin Register (NTR).
**Table S12**. Associations between number of cigarettes smoked per day and decaffeinated coffee (users versus non‐users) in smokers in the Netherlands Twin Register (NTR).
**Figure S1.** NTR = Netherlands Twin Register; CPD = cigarettes per day. The number of participants for each analysis was 1282 for total caffeine, 1790 for coffee, 717 for tea and 402 for cola. Energy drinks were not included due to the low number of users (*n* = 50). Adjusted for age (continuous), educational attainment (continuous) and gender (0 = male 1 = female) and family clustering
**Figure S2.** ALSPAC = Avon Longitudinal Study of Parents and Children; CPD = cigarettes per day. The number of participants for each analysis was 1408 for total caffeine, 993 for coffee, 1125 for tea and 877 for cola. Adjusted for age, educational attainment and social class (all continuous)
**Figure S3.** ALSPAC = Avon Longitudinal Study of Parents and Children; CPD = cigarettes per day. The number of participants for each analysis was 1464 for total caffeine, 1020 for coffee, 1219 for tea and 603 for cola. Adjusted for age, educational attainment and social class (all continuous)
**Figure S4.** ALSPAC = Avon Longitudinal Study of Parents and Children; CPD = cigarettes per day. The number of participants for each analysis was 1499 for total caffeine, 1031 for coffee, 1374 for tea and 815 for cola. Adjusted for age, educational attainment and social class (all continuous)
**Figure S5.** ALSPAC = Avon Longitudinal Study of Parents and Children; CPD = cigarettes per day. The number of participants for each analysis was 1085 for total caffeine, 810 for coffee, 989 for tea and 360 for cola. Adjusted for age, educational attainment and social class (all continuous)
**Figure S6.** ALSPAC = Avon Longitudinal Study of Parents and Children; CPD = cigarettes per day. The number of participants for each analysis was 1121 for total caffeine, 804 for coffee, 872 for tea and 633 for cola. Adjusted for age, educational attainment and social class (all continuous)
**Figure S7.** ALSPAC = Avon Longitudinal Study of Parents and Children; CPD = cigarettes per day. The number of participants for each analysis was 914 for total caffeine, 674 for coffee, 740 for tea and 558 for cola. Adjusted for age, educational attainment and social class (all continuous)
**Figure S8.** ALSPAC = Avon Longitudinal Study of Parents and Children; CPD = cigarettes per day. The number of participants for each analysis was 559 for total caffeine, 586 for coffee, 541 for tea and 414 for cola. Adjusted for age, educational attainment and social class (all continuous)

Supporting info item
