# Supplementary material for: Associations between smoking and caffeine consumption in two European cohorts
Source: Addiction. 2016 Mar 27;111(6):1059–68. doi: 10.1111/add.13298 (PMC4879503; doi:10.1111/add.13298)
Supplement: Supplementary file 1 — Supporting info item [file ADD-111-1059-s001.docx]

**Supplement to manuscript: Associations between smoking and caffeine consumption in two large European cohorts**

Associations between smoking initiation/smoking persistence/smoking heaviness and daily caffeine consumption in ALSPAC______________2-4

Associations between smoking initiation/smoking persistence/smoking heaviness and decaffeinated coffee consumption in ALSPAC__________5

Associations between smoking initiation/smoking persistence/smoking heaviness and daily caffeine consumption in NTR___________________6

Associations between smoking initiation/smoking persistence/smoking heaviness and decaffeinated coffee consumption in NTR_____________7

Associations between smoking heaviness in categories and daily caffeine consumption in NTR_________________________________________8

Associations between smoking heaviness in categories and daily caffeine consumption in ALSPAC ___________________________________9-15

**Table S1**. Associations between smoking initiation (ever versus never smokers) and daily caffeine consumption (in mg) in the *Avon Longitudinal Study of Parents and Children (ALSPAC)*

|  | **Total caffeine – β (95% CI)** | | | | | | | | | | | | | |
| --- | --- | --- | --- | --- | --- | --- | --- | --- | --- | --- | --- | --- | --- | --- |
|  | **18w gestation** | | **32w gestation** | | **2 months** | | **47 months** | | **85 months** | | **97 months** | | **145 months** | |
|  | Unadj | Adj | Unadj | Adj | Unadj | Adj | Unadj | Adj | Unadj | Adj | Unadj | Adj | Unadj | Adj |
| *N* | *12,566* | *9,441* | *10,353* | *8,628* | *7,485* | *5,932* | *8,777* | *7,117* | *7,701* | *6,283* | *6,947* | *5,831* | *4,141* | *3,583* |
| Never smokers | Ref | Ref | Ref | Ref | Ref | Ref | Ref | Ref | Ref | Ref | Ref | Ref | Ref | Ref |
| Ever smokers | 70.9  (65.3 to 76.5) | 55.4  (49.4 to 61.5) | 70.6  (64.8 to 76.4) | 53.9  (47.8 to 60.1) | 66.9  (58.8 to 75.0) | 54.0  (45.1 to 62.9) | 66.2  (59.1 to 73.2) | 59.5  (51.8 to 67.2) | 68.6  (61.0 to 76.2) | 59.0  (50.7 to 67.4) | 63.1  (55.2 to 71.0) | 53.2  (44.7 to 61.8) | 50.1  (40.6 to 59.6) | 44.5  (34.3 to 54.7) |
|  | **Coffee – β (95% CI)** | | | | | | | | | | | | | |
| *N* | *12,689* | *9,500* | *10,815* | *8,990* | *7,873* | *6,187* | *9,006* | *7,278* | *7,901* | *6,427* | *7,185* | *6,021* | *4,730* | *4,039* |
| Never smokers | Ref | Ref | Ref | Ref | Ref | Ref | Ref | Ref | Ref | Ref | Ref | Ref | Ref | Ref |
| Ever smokers | 45.8  (40.9 to 50.6) | 38.7  (33.4 to 44.1) | 44.1  (39.2 to 48.9) | 35.6  (30.3 to 40.9) | 51.3  (43.9 to 58.7) | 39.7  (31.7 to 47.7) | 51.1  (44.4 to 57.9) | 47.3  (39.8 to 54.8) | 55.8  (48.2 to 63.3) | 47.7  (39.3 to 56.0) | 55.5  (47.7 to 63.3) | 48.8  (40.3 to 57.2) | 47.7  (39.1 to 56.4) | 39.9  (30.7 to 49.1) |
|  | **Tea – β (95% CI)** | | | | | | | | | | | | | |
| *N* | *12,674* | *9,499* | *10,877* | *9,045* | *8,140* | *6,354* | *8,989* | *7,277* | *7,892* | *6,427* | *7,165* | *6,004* | *5,752* | *4,909* |
| Never smokers | Ref | Ref | Ref | Ref | Ref | Ref | Ref | Ref | Ref | Ref | Ref | Ref | Ref | Ref |
| Ever smokers | 20.1  (16.7 to 23.5) | 15.4  (11.6 to 19.1) | 24.2  (20.6 to 27.9) | 16.7  (12.8 to 20.6) | 15.3  (10.8 to 19.8) | 12.2  (7.1 to 17.3) | 14.7  (10.3 to 19.1) | 12.3  (7.5 to 17.1) | 12.0  (7.3 to 16.7) | 10.9  (5.7 to 16.0) | 8.9  (4.1 to 13.7) | 5.2  (0.1 to 10.3) | 8.9  (3.5 to 14.2) | 8.6  (2.8 to 14.3) |
|  | **Cola – β (95% CI)** | | | | | | | | | | | | | |
| *N* | *12,639* | *9,473* | *10,720* | *8,921* | *7,973* | *6,235* | *8,934* | *7.233* | *7,825* | *6,371* | *7,076* | *5,929* | *6,198* | *5,220* |
| Never smokers | Ref | Ref | Ref | Ref | Ref | Ref | Ref | Ref | Ref | Ref | Ref | Ref | Ref | Ref |
| Ever smokers | 4.9  (3.9 to 5.9) | 1.8  (0.8 to 2.8) | 2.7  (2.1 to 3.2) | 1.5  (0.9 to 2.1) | 0.9  (0.5 to 1.3) | 0.7  (0.2 to 1.1) | 1.0  (0.6 to 1.3) | 0.5  (0.2 to 0.9) | 0.6  (0.3 to 1.0) | 0.2  (-0.2 to 0.5) | 0.7  (0.3 to 1.0) | 0.2  (-0.2 to 0.6) | 0.7  (0.4 to 1.1) | 0.4  (0.1 to 0.8) |

Linear regression analyses were performed with total caffeine use, caffeine use through coffee only, caffeine use through tea only or caffeine use through cola only as the dependent variable and smoking initiation (0 = never smoking 1 = ever smoking) as the independent variable. Unadj = unadjusted; adj = adjusted for age, educational attainment & social class (all continuous).

**Table S2**. Associations between smoking persistence (current versus former smokers) and daily caffeine consumption (in mg) in the *Avon Longitudinal Study of Parents and Children (ALSPAC)*

|  | **Total caffeine – β (95% CI)** | | | | | | | | | | | | | |
| --- | --- | --- | --- | --- | --- | --- | --- | --- | --- | --- | --- | --- | --- | --- |
|  | **18w gestation** | | **32w gestation** | | **2 months** | | **47 months** | | **85 months** | | **97 months** | | **145 months** | |
|  | Unadj | Adj | Unadj | Adj | Unadj | Adj | Unadj | Adj | Unadj | Adj | Unadj | Adj | Unadj | Adj |
| *N* | *6,341* | *4,502* | *5,179* | *4,139* | *3,467* | *2,699* | *3,970* | *3,155* | *3,281* | *2,603* | *3,126* | *2,522* | *1,773* | *1,505* |
| Former smokers | Ref | Ref | Ref | Ref | Ref | Ref | Ref | Ref | Ref | Ref | Ref | Ref | Ref | Ref |
| Current smokers | 92.8  (83.7 to 101.8) | 88.1  (77.8 to 98.4) | 108.0  (98.9 to 117.1) | 100.8  (90.7 to 111.0) | 80.7  (67.8 to 93.7) | 81.7  (67.0 to 96.4) | 79.4  (67.8 to 90.9) | 83.2  (70.2 to 96.3) | 82.3  (69.3 to 95.4) | 81.6  (66.8 to 96.4) | 89.9  (76.7 to 103.1) | 85.0  (70.2 to 100.0) | 76.4  (60.5 to 92.4) | 78.5  (60.7 to 96.3) |
|  | **Coffee – β (95% CI)** | | | | | | | | | | | | | |
| *N* | *6,423* | *4,540* | *5,429* | *4,333* | *3,660* | *2,826* | *4,078* | *3,226* | *3,375* | *2,670* | *3,238* | *2,610* | *2,039* | *1,702* |
| Former smokers | Ref | Ref | Ref | Ref | Ref | Ref | Ref | Ref | Ref | Ref | Ref | Ref | Ref | Ref |
| Current smokers | 59.4  (51.3 to 67.5) | 66.4  (57.1 to 75.8) | 68.1  (60.2 to 76.1) | 70.5  (61.5 to 79.5) | 74.1  (61.8 to 86.3) | 77.1  (63.5 to 90.8) | 65.5  (54.2 to 76.9) | 74.3  (61.4 to 87.2) | 70.0  (56.8 to 83.3) | 72.7  (57.8 to 87.7) | 73.8  (60.3 to 87.3) | 72.0  (56.8 to 87.3) | 81.9  (67.0 to 96.8) | 74.4  (57.8 to 91.1) |
|  | **Tea – β (95% CI)** | | | | | | | | | | | | | |
| *N* | *6,404* | *4,534* | *5,462* | *4,364* | *3,774* | *2,896* | *4,077* | *3,233* | *3,359* | *2,658* | *3,221* | *2,597* | *2,355* | *1,961* |
| Former smokers | Ref | Ref | Ref | Ref | Ref | Ref | Ref | Ref | Ref | Ref | Ref | Ref | Ref | Ref |
| Current smokers | 26.0  (20.7 to 31.4) | 19.5  (13.2 to 25.7) | 35.6  (29.8 to 41.4) | 25.9  (19.4 to 32.3) | 14.1  (6.8 to 21.3) | 9.7  (1.2 to 18.1) | 14.0  (6.8 to 21.2) | 9.9  (1.9 to 17.9) | 10.1  (2.2 to 18.0) | 6.5  (-2.3 to 15.3) | 16.9  (9.0 to 24.7) | 14.5  (6.0 to 23.1) | 10.9  (1.8 to 19.9) | 8.5  (-1.3 to 18.3) |
|  | **Cola – β (95% CI)** | | | | | | | | | | | | | |
| *N* | *6,385* | *4,519* | *5,385* | *4,300* | *3,711* | *2,846* | *4,048* | *3,210* | *3,342* | *2,645* | *3,185* | *2,567* | *2,545* | *2,092* |
| Former smokers | Ref | Ref | Ref | Ref | Ref | Ref | Ref | Ref | Ref | Ref | Ref | Ref | Ref | Ref |
| Current smokers | 6.6  (5.0 to 8.2) | 2.6  (0.8 to 4.4) | 3.1  (2.2 to 3.9) | 1.4  (0.4 to 2.3) | 1.7  (1.0 to 2.3) | 0.8  (0.1 to 1.6) | 1.4  (0.9 to 1.9) | 0.8  (0.2 to 1.3) | 1.4  (0.8 to 1.9) | 0.5  (-0.1 to 1.1) | 1.4  (0.8 to 2.0) | 0.7  (0.02 to 1.3) | 1.5  (1.0 to 2.1) | 1.0  (0.4 to 1.7) |

Linear regression analyses were performed with total caffeine use, caffeine use through coffee only, caffeine use through tea only or caffeine use through cola only as the dependent variable and smoking persistence (0 = former smoking 1 = current smoking) as the independent variable. Unadj = unadjusted; adj = adjusted for age, educational attainment & social class (all continuous).

**Table S3**. Associations between number of cigarettes smoked per day and daily caffeine consumption (in mg) in smokers from the *Avon Longitudinal Study of Parents and Children (ALSPAC)*

|  | **Total caffeine – β (95% CI)** | | | | | | | | | | | | | |
| --- | --- | --- | --- | --- | --- | --- | --- | --- | --- | --- | --- | --- | --- | --- |
|  | **18w gestation** | | **32w gestation** | | **2 months** | | **47 months** | | **85 months** | | **97 months** | | **145 months** | |
|  | Unadj | Adj | Unadj | Adj | Unadj | Adj | Unadj | Adj | Unadj | Adj | Unadj | Adj | Unadj | Adj |
| *N* | *2,320* | *1,464* | *2,065* | *1,499* | *1,591* | *1,085* | *1,937* | *1,408* | *1,541* | *1,121* | *1,226* | *914* | *692* | *559* |
| Number of cigarettes | 6.0  (4.7 to 7.0) | 5.2  (3.7 to 6.6) | 6.8  (5.5 to 8.0) | 5.9  (4.3 to 7.2) | 7.0  (5.5 to 8.4) | 6.0  (4.3 to 7.7) | 8.1  (6.8 to 9.4) | 8.4  (6.9 to 10.0) | 7.2  (5.8 to 8.5) | 6.6  (4.9 to 8.2) | 7.1  (5.6 to 8.6) | 7.3  (5.6 to 8.9) | 6.9  (5.0 to 8.8) | 7.2  (5.1 to 9.3) |
|  | **Coffee – β (95% CI)** | | | | | | | | | | | | | |
| *N* | *1,567* | *1,020* | *1,423* | *1,031* | *1,206* | *810* | *1,355* | *993* | *1,096* | *804* | *900* | *674* | *742* | *586* |
| Number of cigarettes | 5.7  (4.3 to 7.0) | 4.8  (3.2 to 6.5) | 5.7  (4.4 to 7.1) | 5.7  (4.1 to 7.3) | 5.9  (4.2 to 7.5) | 5.0  (3.0 to 6.9) | 7.8  (6.3 to 9.3) | 8.5  (6.8 to 10.3) | 8.5  (6.8 to 10.1) | 7.6  (5.6 to 9.6) | 7.3  (5.5 to 9.2) | 7.9  (5.9 to 10.0) | 6.6  (4.7 to 8.5) | 6.4  (4.2 to 8.5) |
|  | **Tea – β (95% CI)** | | | | | | | | | | | | | |
| *N* | *1,946* | *1,219* | *1,899* | *1,374* | *1,471* | *989* | *1,549* | *1,125* | *1,199* | *872* | *981* | *740* | *721* | *570* |
| Number of cigarettes | 3.7  (2.9 to 4.4) | 2.7  (1.8 to 3.6) | 4.2  (3.3 to 5.0) | 2.8  (1.8 to 3.8) | 3.2  (2.4 to 4.1) | 2.5  (1.5 to 3.5) | 3.8  (2.9 to 4.7) | 2.7  (1.7 to 3.8) | 2.4  (1.5 to 3.4) | 1.7  (0.7 to 2.8) | 2.8  (1.8 to 3.7) | 2.0  (1.0 to 3.1) | 1.8  (0.7 to 2.8) | 1.7  (0.6 to 2.8) |
|  | **Cola – β (95% CI)** | | | | | | | | | | | | | |
| *N* | *984* | *603* | *1,146* | *815* | *555* | *360* | *1,223* | *877* | *888* | *633* | *756* | *558* | *538* | *414* |
| Number of cigarettes | 0.5  (0.1 to 0.9) | 0.3  (-0.2 to 0.8) | 0.4  (0.2 to 0.5) | 0.1  (-0.1 to 0.4) | 0.2  (-0.0 to 0.4) | 0.1  (-0.1 to 0.3) | 0.1  (-0.0 to 0.2) | 0.1  (-0.0 to 0.2) | 0.1  (0.1 to 0.2) | 0.1  (0.0 to 0.2) | 0.1  (-0.0 to 0.2) | 0.1  (-0.1 to 0.2) | 0.1  (0.0 to 0.2) | 0.1  (-0.0 to 0.2) |

Linear regression analyses were performed with total caffeine use, caffeine use through coffee only, caffeine use through tea only or caffeine use through cola only as the dependent variable and number of cigarettes as the independent variable. Non-caffeine users (or non-users of a specific beverage when analysed individually) were excluded. Unadj = unadjusted; adj = adjusted for age, educational attainment & social class (all continuous).

**Table S4**. Associations between smoking initiation (ever versus never smokers) and decaffeinated coffee consumption (users versus non-users) in the *Avon Longitudinal Study of Parents and Children (ALSPAC)*

|  | **Decaf coffee – OR (95% CI)** | | | | | | | | | | | | | |
| --- | --- | --- | --- | --- | --- | --- | --- | --- | --- | --- | --- | --- | --- | --- |
|  | **18w gestation** | | **32w gestation** | | **2 months** | | **47 months** | | **85 months** | | **97 months** | | **145 months** | |
|  | Unadj | Adj | Unadj | Adj | Unadj | Adj | Unadj | Adj | Unadj | Adj | Unadj | Adj | Unadj | Adj |
| *N* | *12,677* | *9,485* | *10,440* | *8,712* | *7,502* | *5,953* | *9,021* | *7,292* | *7,063* | *5,767* | *6,448* | *5,428* | *4,659* | *3,981* |
| Never smokers | *Ref* | *Ref* | *Ref* | *Ref* | *Ref* | *Ref* | *Ref* | *Ref* | *Ref* | *Ref* | *Ref* | *Ref* | *Ref* | *Ref* |
| Ever smokers | 0.74  (0.67 to 0.81) | 0.97  (0.87 to 1.08) | 0.82  (0.75 to 0.90) | 0.96  (0.87 to 1.06) | 0.77  (0.69 to 0.86) | 0.94  (0.83 to 1.06) | 0.77  (0.69 to 0.86) | 0.84  (0.74 to 0.94) | 0.7  (-0.09 to 0.04) | 0.74  (0.65 to 0.83) | 0.68  (0.60 to 0.78) | 0.75  (0.66 to 0.87) | 0.71  (0.62 to 0.80) | 0.75  (0.65 to 0.87) |

Logistic regression analyses were performed with decaffeinated coffee consumption (0 = non-user 1 = user) as the dependent variable and smoking initiation (0 = never smoking 1 = ever smoking) as the independent variable. Unadj = unadjusted; adj = adjusted for age, educational attainment & social class (all continuous).

**Table S5**. Associations between smoking persistence (current versus former smokers) and decaffeinated coffee consumption (users versus non-users) in the *Avon Longitudinal Study of Parents and Children (ALSPAC)*

|  | **Decaf coffee – OR (95% CI)** | | | | | | | | | | | | | |
| --- | --- | --- | --- | --- | --- | --- | --- | --- | --- | --- | --- | --- | --- | --- |
|  | **18w gestation** | | **32w gestation** | | **2 months** | | **47 months** | | **85 months** | | **97 months** | | **145 months** | |
|  | Unadj | Adj | Unadj | Adj | Unadj | Adj | Unadj | Adj | Unadj | Adj | Unadj | Adj | Unadj | Adj |
| *N* | *6,414* | *4,530* | *5,260* | *4,222* | *3,446* | *2,698* | *4,088* | *3,235* | *3,034* | *2,409* | *2,942* | *2,379* | *1,993* | *1,665* |
| Former smokers | *Ref* | *Ref* | *Ref* | *Ref* | *Ref* | *Ref* | *Ref* | *Ref* | *Ref* | *Ref* | *Ref* | *Ref* | *Ref* | *Ref* |
| Current smokers | 0.56  (0.48 to 0.66) | 0.71  (0.58 to 0.86) | 0.69  (0.60 to 0.79) | 0.78  (0.67 to 0.92) | 0.58  (0.49 to 0.69) | 0.73  (0.59 to 0.89) | 0.70  (0.59 to 0.83) | 0.77  (0.63 to 0.93) | 0.73  (0.60 to 0.88) | 0.78  (0.62 to 0.97) | 0.75  (0.62 to 0.92) | 0.80  (0.63 to 1.01) | 0.62  (0.50 to 0.76) | 0.56  (0.44 to 0.72) |

Logistic regression analyses were performed with decaffeinated coffee consumption (non-user = 0 user = 1) as the dependent variable and smoking persistence (0 = former smoking 1 = current smoking) as the independent variable. Unadj = unadjusted; adj = adjusted for age, educational attainment & social class (all continuous).

**Table S6.** Associations between number of cigarettes smoked per day and decaffeinated coffee consumption (users versus non-users) in smokers from the *Avon Longitudinal Study of Parents and Children (ALSPAC)*

|  | **Decaf coffee – OR (95% CI)** | | | | | | | | | | | | | |
| --- | --- | --- | --- | --- | --- | --- | --- | --- | --- | --- | --- | --- | --- | --- |
|  | **18w gestation** | | **32w gestation** | | **2 months** | | **47 months** | | **85 months** | | **97 months** | | **145 months** | |
|  | Unadj | Adj | Unadj | Adj | Unadj | Adj | Unadj | Adj | Unadj | Adj | Unadj | Adj | Unadj | Adj |
| *N* | *2,454* | *1,527* | *2,217* | *1,616* | *1,617* | *1,112* | *2,066* | *1,487* | *1,489* | *1,081* | *1,206* | *893* | *850* | *666* |
| Number of cigarettes | 0.97  (0.95 to 0.99) | 0.99  (0.97 to 1.02) | 0.98  (0.96 to 0.99) | 0.99  (0.96 to 1.01) | 0.96  (0.94 to 0.98) | 0.97  (0.95 to 1.29) | 0.97  (0.95 to 0.99) | 0.97  (0.95 to 0.99) | 0.96  (0.94 to 0.98) | 0.98  (0.95 to 1.00) | 0.97  (0.95 to 0.99) | 0.97  (0.95 to 1.00) | 0.99  (0.97 to 1.01) | 1.00  (0.97 to 1.02) |

Logistic regression analyses were performed with decaffeinated coffee consumption (0 = non-user 1 = user) as the dependent variable and number of cigarettes as the independent variable. Unadj = unadjusted; adj = adjusted for age, educational attainment & social class (all continuous).

**Table S7**. Associations between smoking initiation (ever versus never smokers) and daily caffeine consumption (in mg) in the *Netherlands Twin Register (NTR)*

|  | **Total caffeine – β (95% CI)** | | **Coffee – β (95% CI)** | | **Tea – β (95% CI)** | | **Cola – β (95% CI)** | | **Energy drink – β (95% CI)** | |
| --- | --- | --- | --- | --- | --- | --- | --- | --- | --- | --- |
|  | Unadj | Adj | Unadj | Adj | Unadj | Adj | Unadj | Adj | Unadj | Adj |
| *N* | *17,736* | *11,805* | *21,682* | *14,584* | *17,736* | *11,805* | *17,736* | *11,805* | *17,736* | *11,805* |
| Never smokers | Ref | Ref | Ref | Ref | Ref | Ref | Ref | Ref | Ref | Ref |
| Ever smokers | 108.8  (102.6 to 114.9) | 52.8  (45.6 to 60.0) | 120.3  (114.7 to 125.9) | 76.4  (69.7 to 83.0) | -10.3  (-12.7 to -7.9) | -16.1  (-19.1 to -13.1) | 0.3  (-0.2 to 0.7) | 1.2  (0.8 to 1.7) | 1.0  (0.5 to 1.5) | 0.6  (0.2 to 0.9) |

Linear regression analyses were performed with total caffeine use, caffeine use through coffee only, caffeine use through tea only, caffeine use through cola only or caffeine use through energy drinks only as the dependent variable and smoking initiation (0 = never smoking 1 = ever smoking) as the independent variable. Unadj = unadjusted; adj = adjusted for age (continuous), educational attainment (continuous) & gender (0 = male 1 = female); analyses were corrected for family clustering by utilizing the robust cluster option in STATA.

**Table S8.** Associations between smoking persistence (current versus former smokers) and daily caffeine consumption (in mg) in the *Netherlands Twin Register (NTR)*

|  | **Total caffeine – β (95% CI)** | | **Coffee – β (95% CI)** | | **Tea – β (95% CI)** | | **Cola – β (95% CI)** | | **Energy drink – β (95% CI)** | |
| --- | --- | --- | --- | --- | --- | --- | --- | --- | --- | --- |
|  | Unadj | Adj | Unadj | Adj | Unadj | Adj | Unadj | Adj | Unadj | Adj |
| *N* | *7,088* | *5,400* | *8,762* | *6,619* | *7,088* | *5,400* | *7,088* | *5,400* | *7,088* | *5,400* |
| Former smokers | Ref | Ref | Ref | Ref | Ref | Ref | Ref | Ref | Ref | Ref |
| Current smokers | 14.1  (3.5 to 24.8) | 57.9  (45.2 to 70.5) | 26.1  (16.4 to 35.7) | 71.2  (59.7 to 82.8) | -19.0  (-22.6 to -15.4) | -17.1  (-21.5 to -12.7) | 4.1  (3.3 to 4.9) | 1.9  (1.0 to 2.8) | 5.9  (4.7 to 7.1) | 1.5  (0.7 to 2.4) |

Linear regression analyses were performed with total caffeine use, caffeine use through coffee only, caffeine use through tea only, caffeine use through cola only or caffeine use through energy drinks only as the dependent variable and smoking persistence (0 = former smoking 1 = current smoking) as the independent variable. Unadj = unadjusted; adj = adjusted for age (continuous), educational attainment (continuous) & gender (0 = male 1 = female); analyses were corrected for family clustering by utilizing the robust cluster option in STATA.

**Table S9 .** Associations between number of cigarettes smoked per day and daily caffeine consumption (in mg) in smokers from the *Netherlands Twin Register (NTR)*

|  | **Total caffeine – β (95% CI)** | | **Coffee – β (95% CI)** | | **Tea – β (95% CI)** | | **Cola – β (95% CI)** | | **Energy drink – β (95% CI)** | |
| --- | --- | --- | --- | --- | --- | --- | --- | --- | --- | --- |
|  | Unadj | Adj | Unadj | Adj | Unadj | Adj | Unadj | Adj | Unadj | Adj |
| *N* | *1,954* | *1,282* | *2,572* | *1,790* | *1,122* | *717* | *751* | *402* | *243* | *50* |
| Number of cigarettes | 6.1 (4.4 to 7.9) | 3.7 (1.9 to 5.5) | 6.5 (5.1 to 7.9) | 5.0 (3.5 to 6.4) | -0.0 (-0.7 to 0.7) | 0.2 (-0.7 to 1.1) | 0.9 (0.6 to 1.3) | 0.8 (0.3 to 1.2) | 2.3 (0.8 to 3.8) | 1.5 (-1.5 to 4.4) |

Linear regression analyses were performed with total caffeine use, caffeine use through coffee only, caffeine use through tea only, caffeine use through cola only or caffeine use through energy drinks only as the dependent variable and number of cigarettes as the independent variable. Non-caffeine users (or non-users of a specific beverage when analysed individually) were excluded. Unadj = unadjusted; adj = adjusted for age (continuous), educational attainment (continuous) & gender (0 = male 1 = female); analyses were corrected for family clustering by utilizing the robust cluster option in STATA.

**Table S10.** Associations between smoking initiation (ever versus never smokers) and decaffeinated coffee consumption (users versus non-users) in the *Netherlands Twin Register (NTR)*

|  | **Decaf coffee – OR (95% CI)** | |
| --- | --- | --- |
|  | Unadj | Adj |
| *N* | *17,736* | *11,805* |
| Never smokers | *Ref* | *Ref* |
| Ever smokers | 1.43 (1.29 to 1.58) | 0.94 (0.80 to 1.06) |

Logistic regression analyses were performed with decaffeinated coffee consumption (0 = non-user 1 = user) as the dependent variable and smoking initiation (0 = never smoking 1 = ever smoking) as the independent variable. Unadj = unadjusted; adj = adjusted for age (continuous), educational attainment (continuous) & gender (0 = male 1 = female); analyses were corrected for family clustering by utilizing the robust cluster option in STATA.

**Table S11.** Associations between smoking persistence (current versus former smokers) and decaffeinated coffee consumption (users versus non users) in the *Netherlands Twin Register (NTR)*

|  | **Decaf coffee – OR (95% CI)** | |
| --- | --- | --- |
|  | Unadj | Adj |
| *N* | *7,088* | *5,400* |
| Former smokers | *Ref* | *Ref* |
| Current smokers | 0.58 (0.49 to 0.69) | 0.80 (0.66 to 0.98) |

Logistic regression analyses were performed with decaffeinated coffee consumption (0 = non-user 1 = user) as the dependent variable and smoking persistence (0 = former smoking 1 = current smoking) as the independent variable; unadj = unadjusted; adj = adjusted for age (continuous), educational attainment (continuous) & gender (0 = male 1 = female); analyses were corrected for family clustering by utilizing the robust cluster option in STATA.

**Table S12**. Associations between number of cigarettes smoked per day and decaffeinated coffee (users versus non users) in smokers in the *Netherlands Twin Register (NTR)*

|  | **Decaf coffee – OR (95% CI)** | |
| --- | --- | --- |
|  | Unadj | Adj |
| *N* | *1,999* | *1,308* |
| Number of cigarettes | 0.99 (0.97 to 1.01) | 0.98 (0.96 to 1.01) |

Logistic regression analyses were performed with decaffeinated coffee consumption (0 = non-user 1 = user) as the dependent variable and number of cigarettes as the independent variable. Unadj = unadjusted; adj = adjusted for age (continuous), educational attainment (continuous) & gender (0 = male 1 = female); analyses were corrected for family clustering by utilizing the robust cluster option in STATA.

**
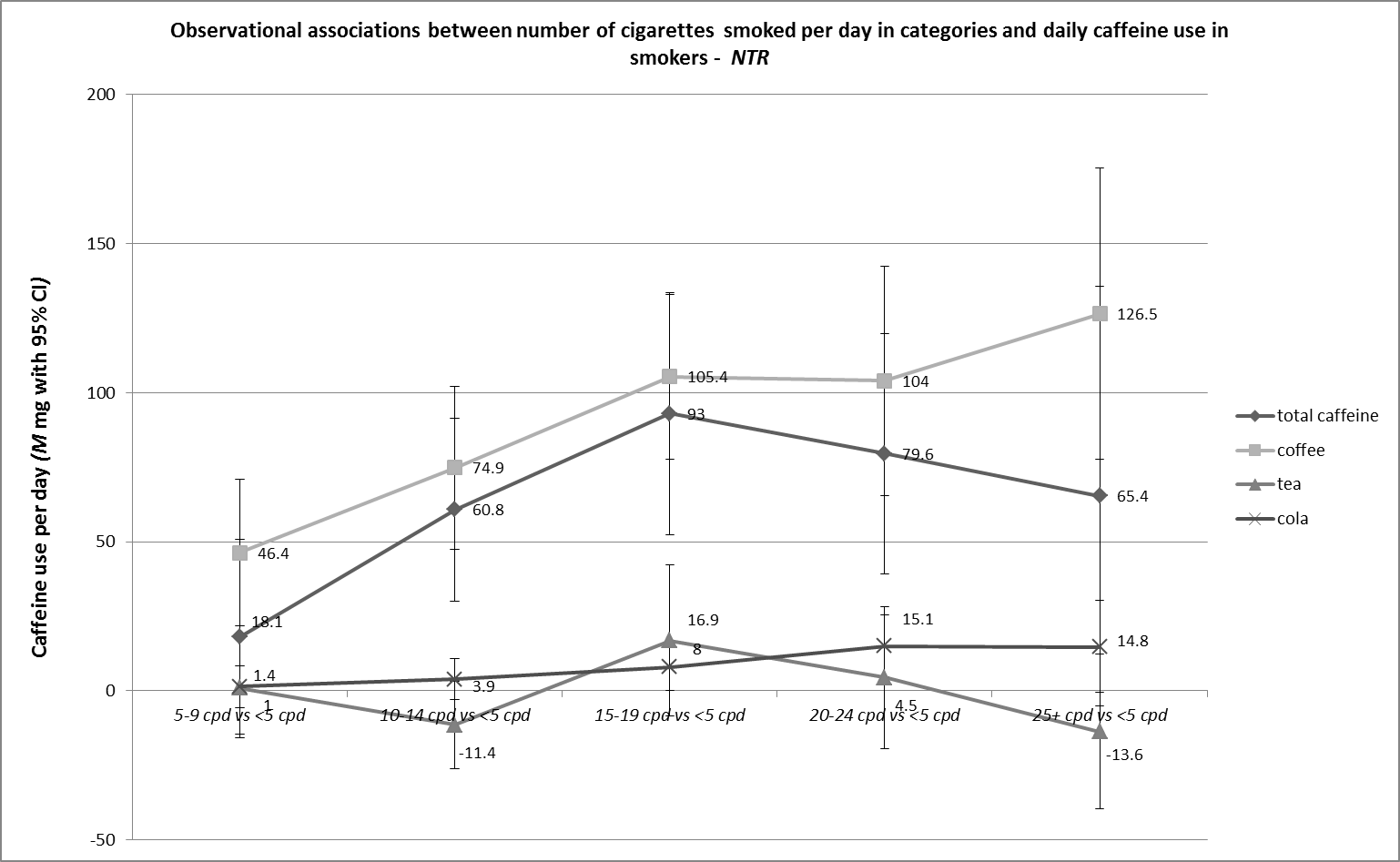
**

**Figure S1.** NTR = Netherlands Twin Register; cpd = cigarettes per day. The number of participants for each analysis was 1,282 for total caffeine, 1,790 for coffee, 717 for tea and 402 for cola. Energy drinks were not included due to the low number of users (n=50). Adjusted for age (continuous), educational attainment (continuous) & gender (0 = male 1 = female) and family clustering.

**
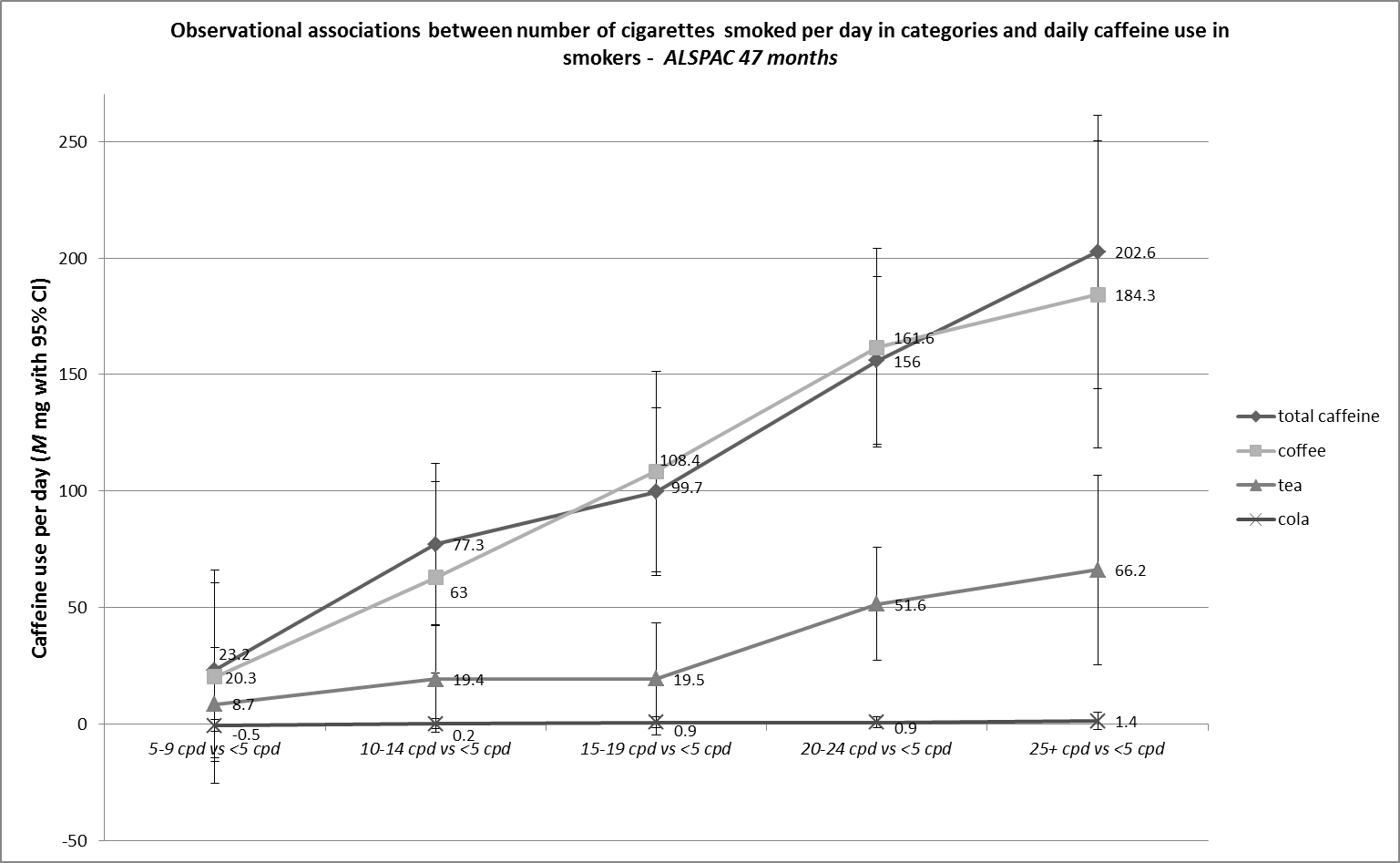
**

**Figure S2.** ALSPAC = Avon Longitudinal Study of Parents and Children; cpd = cigarettes per day. The number of participants for each analysis was 1,408 for total caffeine, 993 for coffee, 1,125 for tea and 877 for cola. Adjusted for age, educational attainment & social class (all continuous).


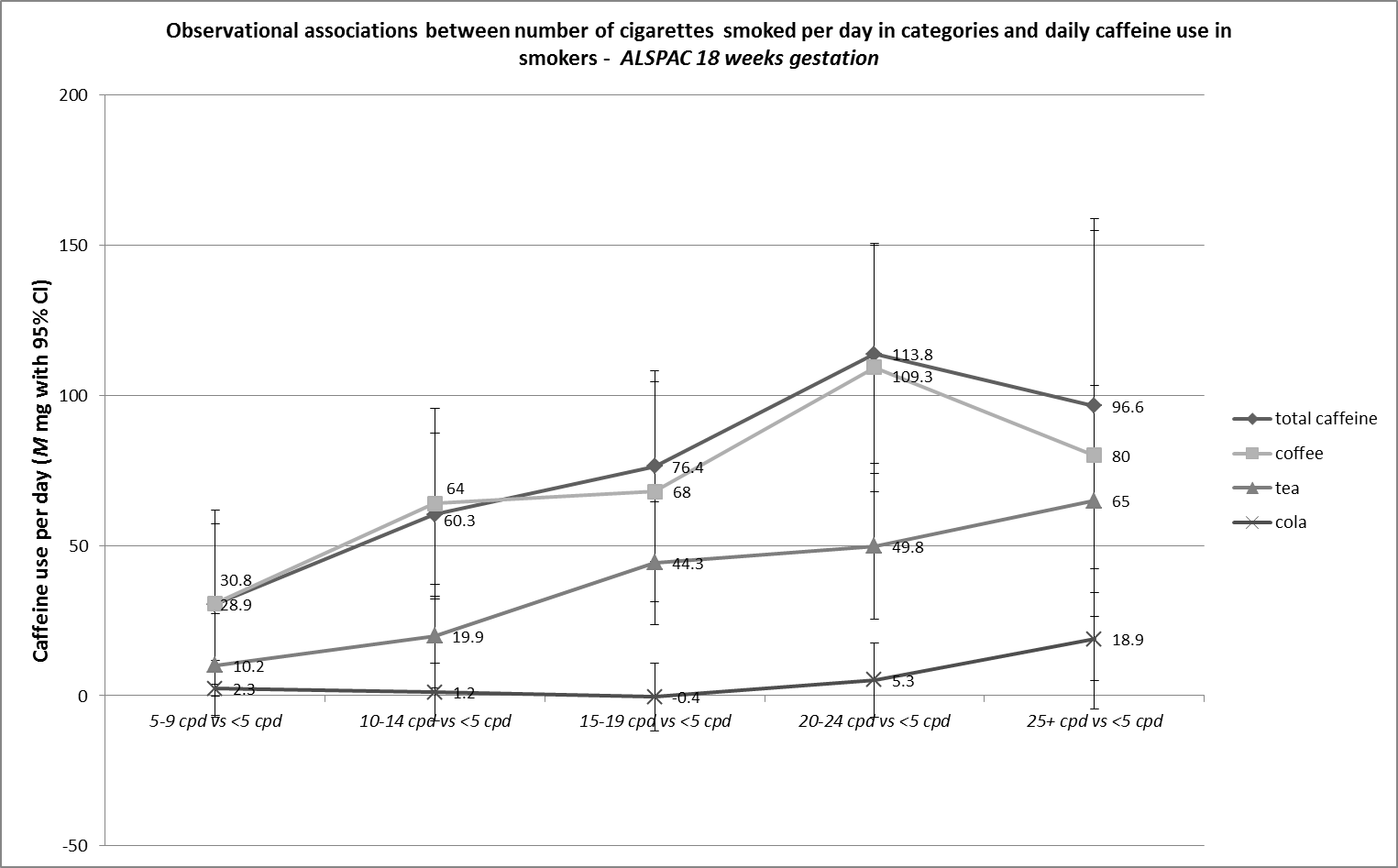


**Figure S3.** ALSPAC = Avon Longitudinal Study of Parents and Children; cpd = cigarettes per day. The number of participants for each analysis was 1,464 for total caffeine, 1,020 for coffee, 1,219 for tea and 603 for cola. Adjusted for age, educational attainment & social class (all continuous).

**
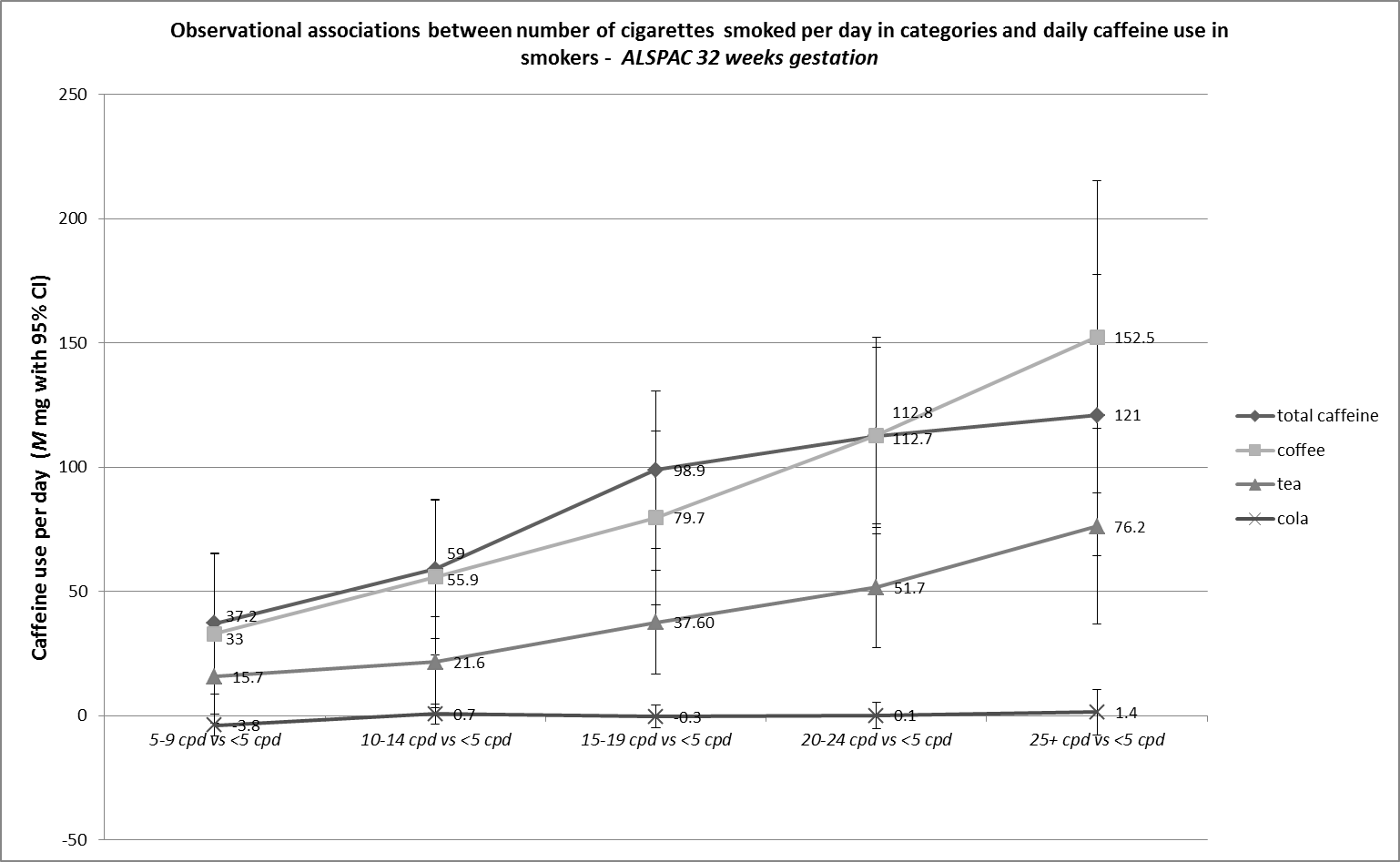
**

**Figure S4.** ALSPAC = Avon Longitudinal Study of Parents and Children; cpd = cigarettes per day. The number of participants for each analysis was 1,499 for total caffeine, 1,031 for coffee, 1,374 for tea and 815 for cola. Adjusted for age, educational attainment & social class (all continuous).

**
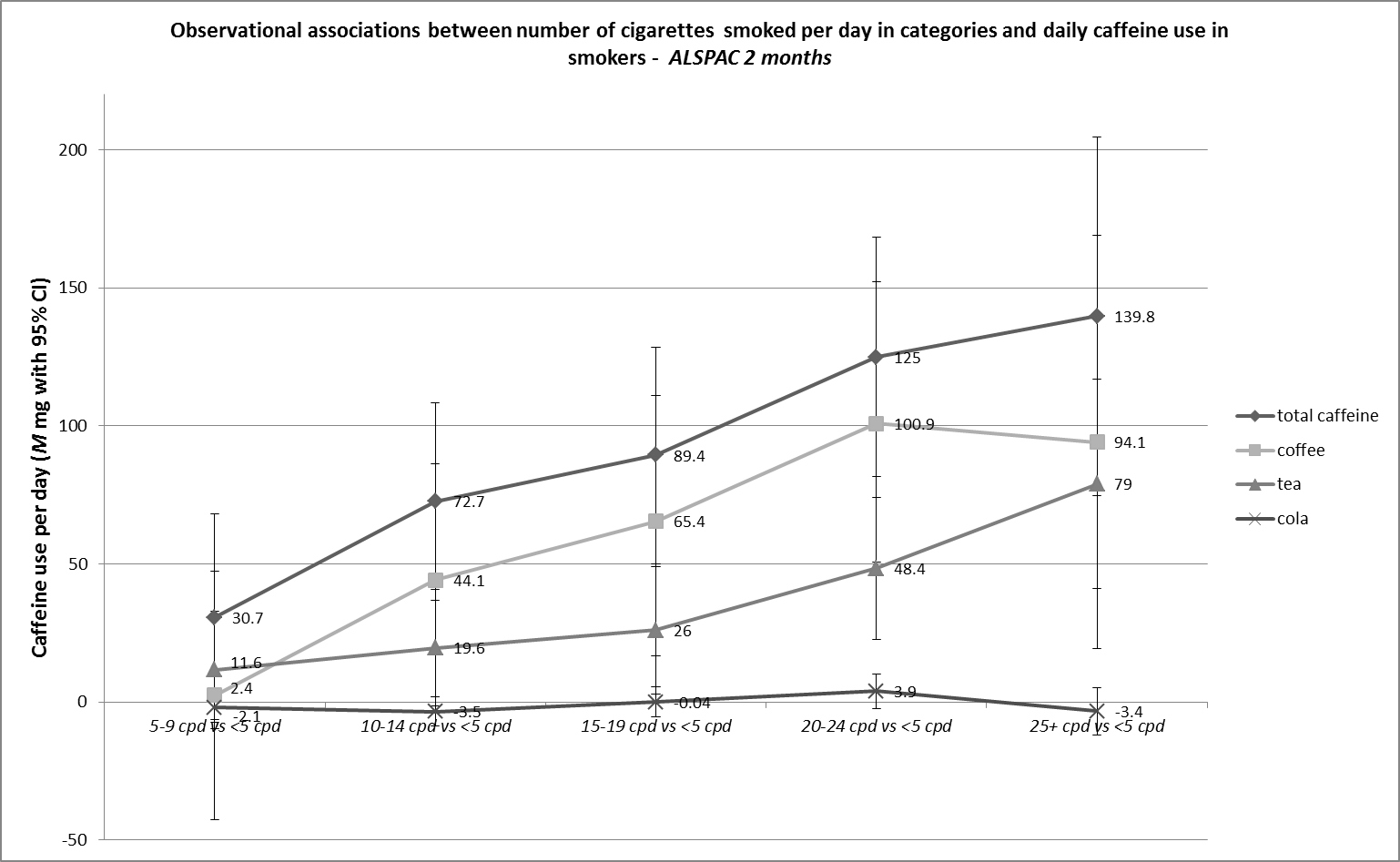
**

**Figure S5.** ALSPAC = Avon Longitudinal Study of Parents and Children; cpd = cigarettes per day. The number of participants for each analysis was 1,085 for total caffeine, 810 for coffee, 989 for tea and 360 for cola. Adjusted for age, educational attainment & social class (all continuous).

**
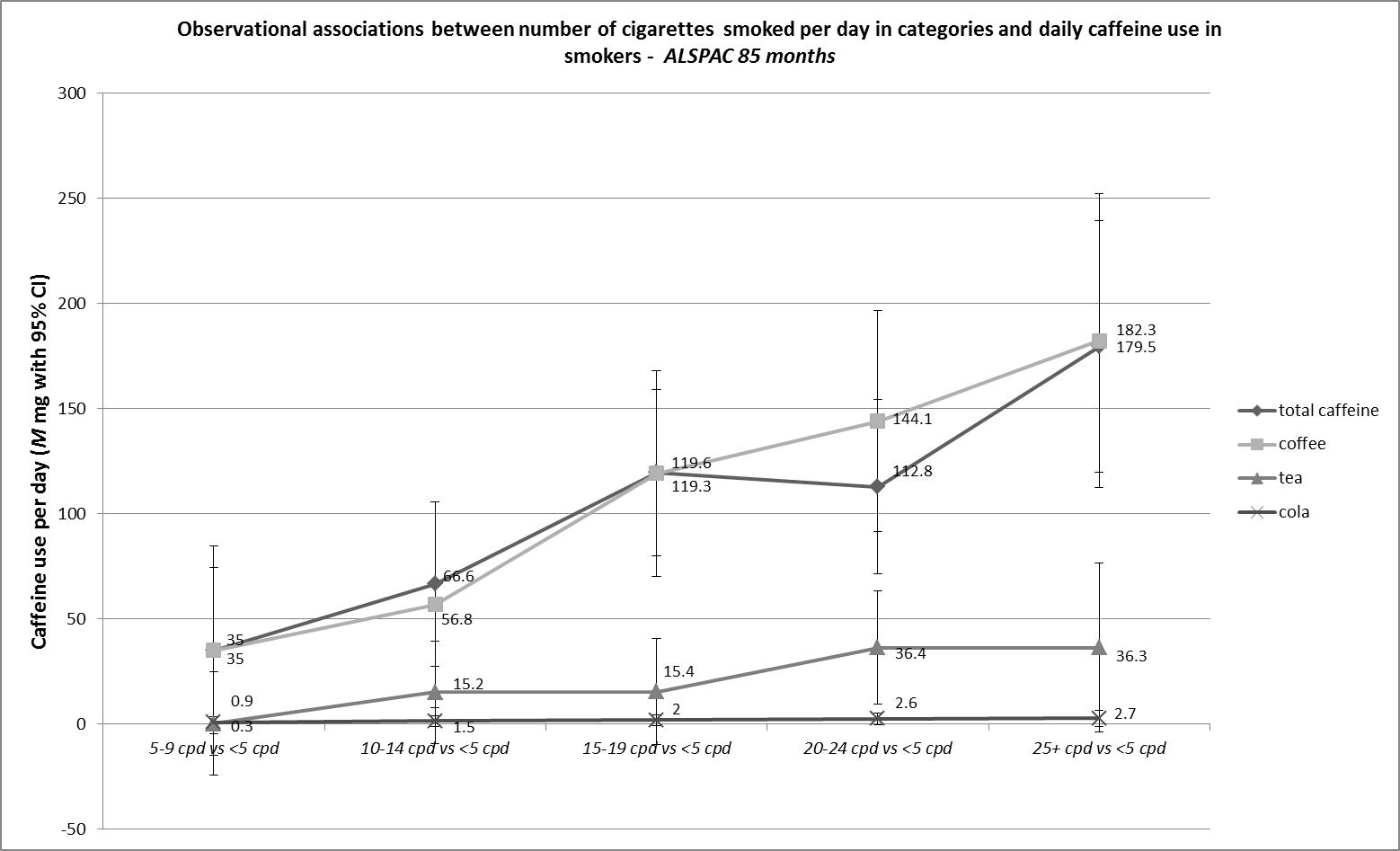
**

**Figure S6.** ALSPAC = Avon Longitudinal Study of Parents and Children; cpd = cigarettes per day. The number of participants for each analysis was 1,121 for total caffeine, 804 for coffee, 872 for tea and 633 for cola. Adjusted for age, educational attainment & social class (all continuous).

**
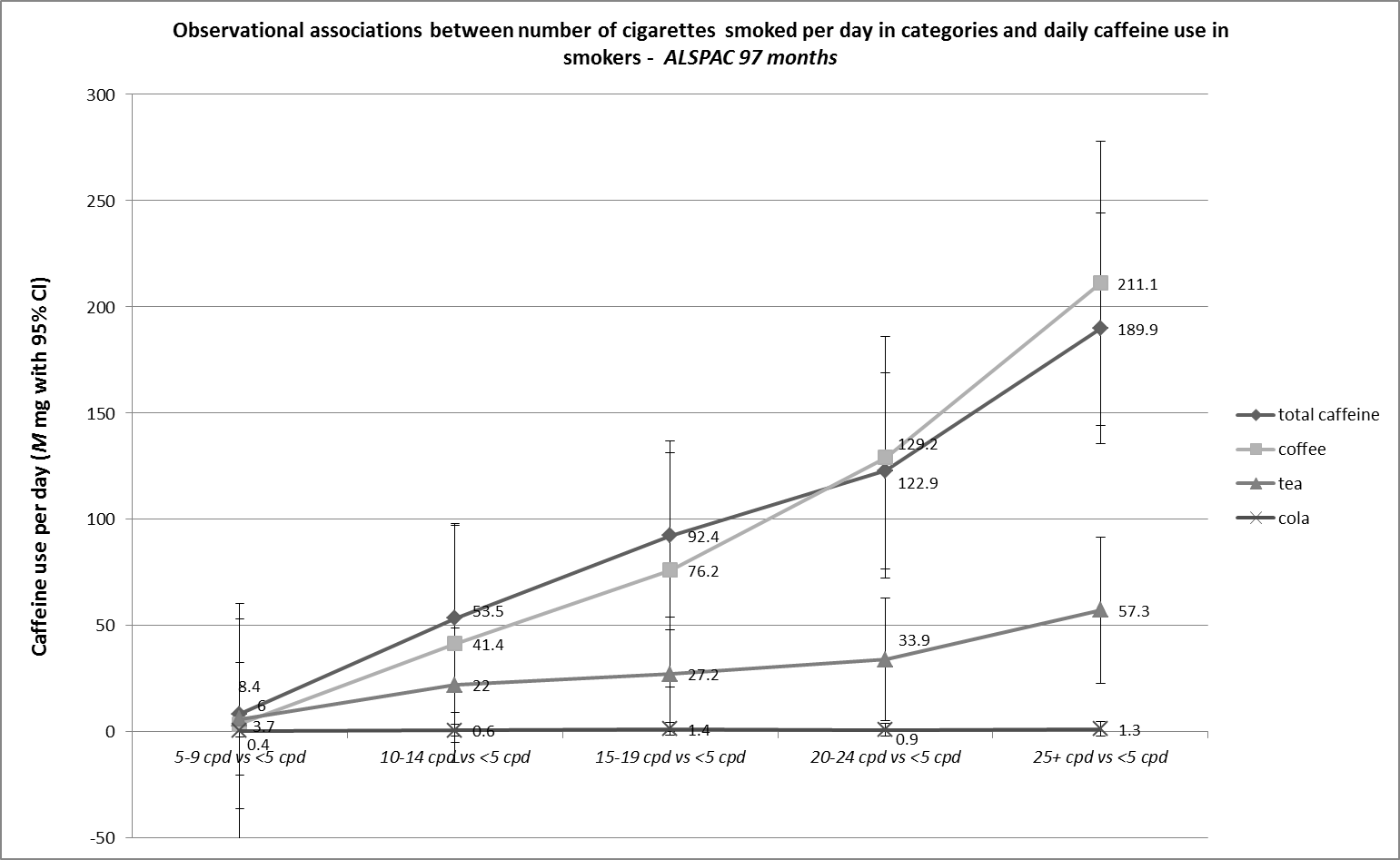
**

**Figure S7.** ALSPAC = Avon Longitudinal Study of Parents and Children; cpd = cigarettes per day. The number of participants for each analysis was 914 for total caffeine, 674 for coffee, 740 for tea and 558 for cola. Adjusted for age, educational attainment & social class (all continuous).


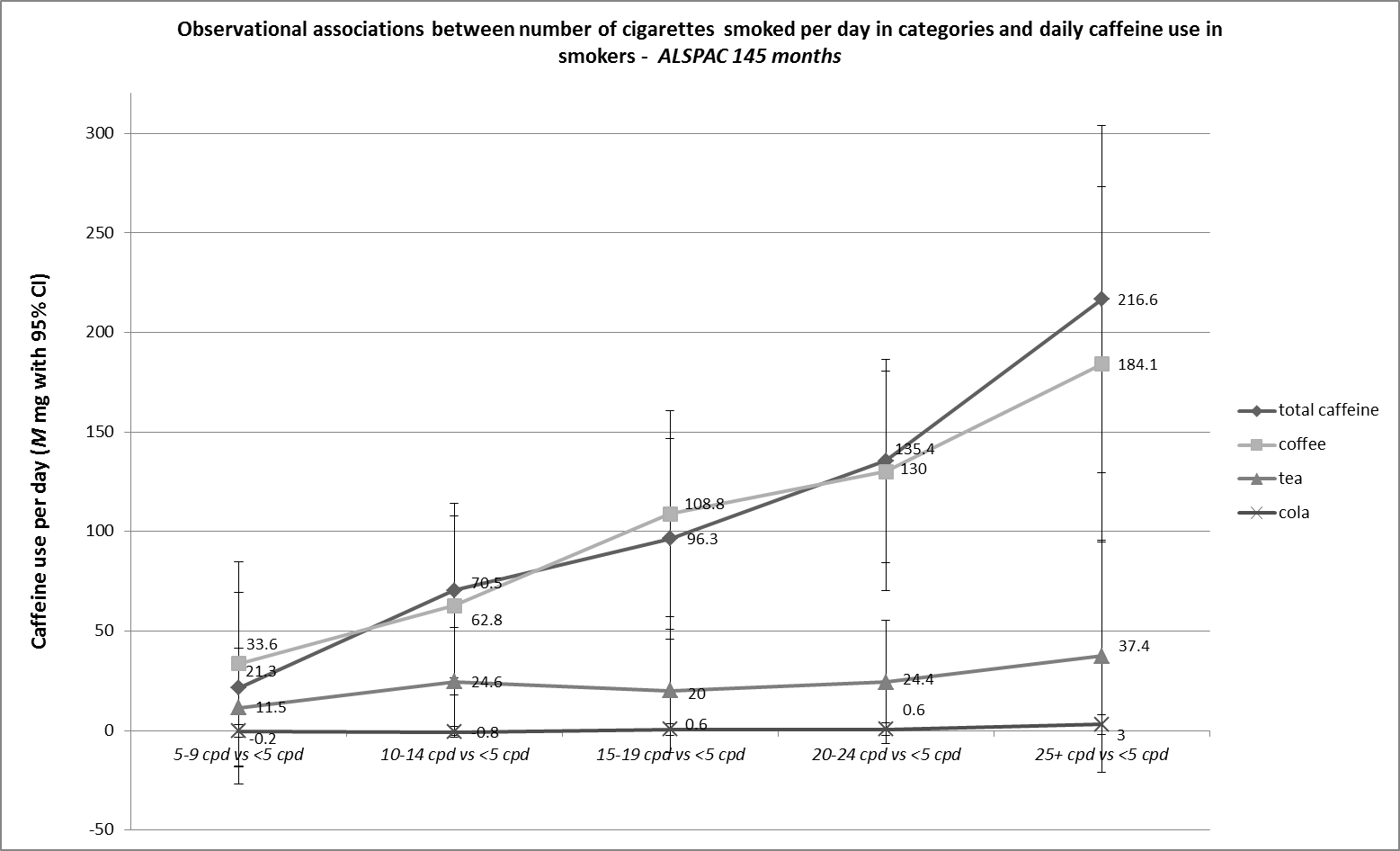


**Figure S8.** ALSPAC = Avon Longitudinal Study of Parents and Children; cpd = cigarettes per day. The number of participants for each analysis was 559 for total caffeine, 586 for coffee, 541 for tea and 414 for cola. Adjusted for age, educational attainment & social class (all continuous).
